# Supplementary material for: Effectiveness of therapeutic ultrasound on reducing pain intensity and functional disability in patients with plantar fasciitis: a systematic review of randomised controlled trials
Source: PeerJ. 2024 Mar 22;12:e17147. doi: 10.7717/peerj.17147 (PMC10962347; doi:10.7717/peerj.17147)
Supplement: Supplemental Information 2 [file peerj-12-17147-s002.docx]

- Planter fasciitis is a common musculoskeletal problem affecting a large number of professionals and the middle-aged general population (Buchbinder 2004; Pohl et al. 2009; Rabadi et al. 2022).
- There is a need for the development of the most effective interventions that can help rehabilitation outcomes.
- Ultrasound therapy has the potential for effective management of musculoskeletal problems including planter fasciitis (Baker et al. 2001; Papadopoulos & Mani 2020).
- Previous studies have evaluated the effectiveness of ultrasound therapy for the treatment of various musculoskeletal disorders using different intensities/duration of therapy, outcome measures, and participants' characteristics
- However, the evidence around its effectiveness for the management of plantar fasciitis is still unclear and needs a further review of the current literature.
- This study aims to discuss the effectiveness of therapeutic ultrasound in decreasing pain intensity and improving functional disability in patients with plantar fasciitis.
- Synthesis of evidence around ultrasound therapy for plantar fasciitis can help inform clinicians’ decisions on its use in the treatment of plantar fasciitis and understand contextual factors/intervention protocols that can be used in practice.
